# Protein and Oil Contents, Micro- and Macronutrients, and Other Quality Indicators of Soybean Cultivated in Lowland Fields

**DOI:** 10.3390/foods13233719

**Published:** 2024-11-21

**Authors:** Jéssica Streck Baisch, Mara Grohs, Paulo Ademar Avelar Ferreira, Gustavo Andrade Ugalde, Marcus Vinícius Tres, Giovani Leone Zabot

**Affiliations:** 1 Laboratory of Agroindustrial Processes Engineering (LAPE), Federal University of Santa Maria, 3013 Taufik Germano Rd., Universitário II DC, Cachoeira do Sul 96503-205, Brazil; jessica_baisch@outlook.com (J.S.B.); marcus.tres@ufsm.br (M.V.T.); 2 Rio Grandense Rice Institute (IRGA), 493 Marechal Floriano St, Cachoeira do Sul 96506-750, Brazil; grohs.mara@gmail.com; 3 Campus Cachoeria Do Sul, Federal University of Santa Maria, Cachoeira Do Sul 96506-205, Brazil; paulo.ferreira@ufsm.br; 4 Department of Rural Engineering, Federal University of Santa Maria, 1000 Roraima Av, Santa Maria 97105-900, Brazil; gandradeugalde@yahoo.com.br

**Keywords:** soybean oil, fatty acids, proximate analysis, micronutrients, proteins, macronutrients

## Abstract

The cultivation of soybean is being expanded in traditional areas cultivated with rice, called the lowlands. However, soil characteristics are different from those in the highlands, which influences the exportation of nutrients to the grains. Therefore, this study aimed to determine the physical-chemical and technological characteristics of soybean grains harvested in lowlands in Brazil. Two-year crops (2021/22 and 2022/23) were used with two types of soil preparation (scarified and non-scarified) and six cover crop treatments (oats, clover, ryegrass, fallow, ryegrass + oats, and ryegrass + clover). The influence of these treatments was evaluated in terms of the grain yield, oil and protein contents, oil composition, quality indices (acidity, peroxide, iodine, and saponification), and contents of ash, carbohydrates, and micro- and macronutrients. Grain yield achieved an average of 3829.8 kg ha^−1^. Soil scarification positively influenced grain yield and contributed to higher protein and oil contents, with maximum values of 32.7 wt% and 27.6 wt%, respectively. The main fatty acids in oil were oleic acid (22.13 ± 1.48–26.32 ± 0.98%) and linoleic acid (36.32 ± 1.57–52.18 ± 1.58%). The macronutrients phosphorus (5.12 ± 0.39–5.79 ± 0.37 kg ton^−1^), calcium (2.79 ± 0.19–3.05 ± 0.18 kg ton^−1^), magnesium (2.37 ± 0.14–2.57 ± 0.13 kg ton^−1^), and sulfur (2.85 ± 0.18–3.19 ± 0.20 kg ton^−1^), and the micronutrients copper (9.73 ± 1.42–11.68 ± 1.07 g ton^−1^), iron (111.42 ± 6.86–122.02 ± 5.00 g ton^−1^), and manganese (43.58 ± 3.34–47.08 ± 2.74 g ton^−1^) were in agreement with the values reached in the highlands. For potassium (18.87 ± 0.38–29.29 ± 1.44 kg ton^−1^) and zinc (30.02 ± 2.45–38.00 ± 1.03 g ton^−1^), soil scarification allows higher levels of absorption. The use of ryegrass as a cover crop allows higher levels of nitrogen absorption, reaching up to 44.93 ± 2.74 kg ton^−1^. Regarding the acidity (0.19–0.52%), peroxide (9.64–16.39 mEq O_2_ kg^−1^), iodine (85.34–91.91 mg KI g^−1^), and saponification (182.33–203.74 mg KOH g^−1^) indices of the oil, all values were obtained in accordance with the scientific literature. The conclusions of this study indicate that it is possible to cultivate soybean in lowlands after developing the proper soil preparation. Consequently, the yields are increased, and grains will benefit from higher protein and oil contents, enhancing soybean quality for commercialization.

## 1. Introduction

Soybean is an important crop used for animal and human consumption as a source of oil and high-quality protein [1]. This crop contributes to global food security because it achieves approximately 27% of the production and supply of vegetable oils in the global market [2]. Soybean grains are highly nutritious because they supply proteins, carbohydrates, dietary fiber, vitamins, minerals, bioactive compounds, and fatty acids [3]. The suitability of soybean oil for specific uses is determined by the fatty acid composition from which various indices and indicators can be calculated. Soybean oil is used in foods such as dessert batter, salad oil, and mayonnaise, among others [4].

Soybean contains approximately 40% protein and 20% oil. It is used to produce natural surfactants, protein products, lecithin, vegetable oil, and polysaccharides [5]. It is a highly important food ingredient and a vital source of nutrients rich in isoflavones. Soybean is also used as a component in fermented foods, pastes, and soy sauces. It is consumed in the form of processed products such as soy milk and soy curd. Soybean is the main source of protein in countries where rice is the staple diet due to its high content of the essential amino acid lysine [6].

The quality of soybean is an important criterion for its commercialization and processing. However, the characteristics of the soil in lowland fields traditionally used for rice crops, such as low friability and excess water, influence the absorption of nutrients for soybean crops. Consequently, it is a challenge to introduce soybean cultivation in lowland fields [7]. Therefore, adapting management practices is a necessity for its sustainable agricultural production in lowland fields with poor soil quality. The use of cover crops as a management practice has been adopted in many no-till systems. Direct seeding maintains the soil moisture status by influencing evaporation, runoff, and runoff after rainfall events. The inclusion of cover crops in a no-till production system improves the soil’s physical properties and microbial enzyme activity [8].

In the study carried out by Ody et al. [7], the oil, protein, and micro- and macronutrient contents of soybean sown in Brazilian lowlands in November and December in the crop years 2018/2019 and 2021/2022, with and without soil scarification, and different fertilization levels, were evaluated. That study concluded that sowing earlier is favorable to obtain more protein and oil. Also, it is not necessary to use high fertilization levels because the medium fertilization level is sufficient. However, there is a gap in this area because no study has been developed with different soil coverages by plants during the off-season period.

Consequently, the physical-chemical characteristics of soybean grains from lowland fields are still not well known, and further studies on this subject are needed. Overall, the objective is to improve the quality and grain yield of soybean in the lowlands by using the right management practices and technologies. In this context, this work addresses this issue by presenting the physical-chemical and technological characteristics of soybean grains obtained from different treatments in Brazilian lowland fields. The influences of the type of soil preparation and the different varieties of cover crops were evaluated based on their protein and oil contents, fatty acids in the oil, micro- and macronutrients, and acidity, peroxide, iodine, and saponification indices of the oil.

## 2. Materials and Methods

### 2.1. Experiments in the Lowland Fields

The experiments in the lowland fields were carried out at the Regional Research Station of the Instituto Rio Grandense do Arroz (IRGA), in Cachoeira do Sul (Brazil), located at the following geographic coordinates: latitude 29°43′23″ south and longitude 53°43′15″ west, with an altitude of approximately 95 m above sea level. The soil used is classified as Hydromorphic Eutrophic Arenic Planosol, with the following characteristics: pH = 5.6; P = 7.3 mg dm^−3^; K = 39 mg dm^−3^; and organic matter = 13 g kg^−1^.

The treatments consisted of sowing the following soil cover crops in the Brazilian autumn–winter: ryegrass, black oats, clover, ryegrass + clover, and ryegrass + black oats. The control treatment consisted of fallow (Table 1). The soil was scarified (depth of 25 cm) or non-scarified in strips, and within each strip the cover crops were randomly distributed in a randomized block design, representing a two-factorial design (5 × 2), with four replicates. In total, there were 40 experimental units of 25 m^2^ (5 × 5 m).

The scarification of the strips was carried out one month before soybean sowing using a four-shank scarifier. The cover crops were sown six months before soybean sowing. The cover crops sowing was carried out with a plot seeder, with nine rows, spaced 0.17 m apart. Ryegrass was sown at a density of 15 kg ha^−1^, using the Ponteio cultivar. Black oat was sown at a density of 80 kg ha^−1^. Clover was sown at a density of 8 kg ha^−1^. In the black oat and ryegrass consortia, 50 and 10 kg ha^−1^ of each crop were used, respectively. In the ryegrass and Persian clover consortia, 10 and 5 kg ha^−1^ of each crop were used, respectively. The fallow treatment was kept desiccated throughout the autumn–winter period.

When the cover crops were sown, 250 kg ha^−1^ of fertilizer of the formula of 04-17-27 (NPK) was used, totaling 10, 42.5, and 67.5 kg ha^−1^ of N, P_2_O_5_, and K_2_O, respectively. Thirty days after cover crop emergence, another 50 kg ha^−1^ of N was spread with the formula of 45-00-00 (NPK) only in the grass plots, and 100 kg ha^−1^ of mono-ammonium phosphate (MAP 11-52-00) was spread in the Persian clover plots. All other phytosanitary treatments were carried out according to the needs of each crop. One month before soybean sowing, the area was desiccated using 2170 g ha^−1^ of glyphosate (Zap Qi^®^), associated with 100 g ha^−1^ of saflufenacil (Heat^®^) and 0.5% (*v*/*v*) of mineral oil. The parameters related to the physical part of the soil were measured before soybean sowing and after grain harvesting. Soil density was quantified using the volumetric cylinder method, at depths of 0–5, 5–10, 10–20, and 20–30 cm.

The soybean cultivar Neo 610 IPRO (GM 6.1), with a medium cycle (135 days), and an indeterminate growth habit, with the sowing of 28.6 plants m^2^, was used. Base fertilization for the soybean was carried out with 350 kg ha^−1^ of 05-30-15 (NPK). When the plants were at the V4 stage, 100 kg ha^−1^ of K was spread in the form of potassium chloride (KCl 00-00-60). Weed control was performed in the pre-emergence of the crop, through the application of 1.2 L ha^−1^ of the herbicide s-metolachlor (Dual Gold^®^), 2940 g ha^−1^ of glyphosate (Round Transorb^®^), 80 mL ha^−1^ of lambda-cyhalothrin + chlorantraniliprole (Ampligo^®^, Syngenta, Basel, Switzerland), 1.5 kg ha^−1^ of sodium octaborate, and 0.5% (*v*/*v*) of mineral oil. In post-emergence, the herbicide glyphosate was used, and the applications of fungicide and insecticide were carried out as needed. The following two harvests were tested: 2021/2022 and 2022/2023.

Soybean grain productivity was measured by harvesting two central rows, four meters long, of each experimental unit, totaling 4 m^2^. The material was subjected to the threshing, cleaning, drying, and weighing of the harvested grains. After all these procedures, for all treatments, the samples (a total of 80 samples) were stored in bags and transported to the laboratory for analysis.

### 2.2. Analyses of Quality Indicators

The analyses focused on grain moisture, oil yield, oil composition in terms of fatty acids, acidity, peroxide, iodine and saponification indices, total protein, ash, carbohydrate, micronutrients, and macronutrients.

#### 2.2.1. Moisture

Each sample (5 g) was placed in Petri dishes and placed in a drying oven (80/150, Lucadema, São José do Rio Preto, Brazil) at 105 °C. The samples were subjected to mass determination when they reached a constant mass (approximately 24 h) on an analytical balance (M214Ai, Bel, Piracicaba, Brazil).

#### 2.2.2. Oil

Oil extraction was performed according to the methodology used by Confortin et al. [9]. Each sample (5 g) was placed on a filter paper cartridge and 150 mL of n-hexane (Dynamic, 98.5%) was added to a round-bottom flask. The extraction was carried out for six hours in a Soxhlet apparatus. Afterward, the n-hexane was separated from the oil in a rotary evaporator (SKL-25a, Even, São Paulo, Brazil). The mass was measured on an analytical balance (M214Ai, Bel, Piracicaba, Brazil). Four replicates were performed for each sample to obtain an average ± standard deviation.

#### 2.2.3. Oil Composition

The samples of the extracted oil were subjected to the methylation step. A quantity of 250 μL of the internal standard methyl tricosanoate was added in a 4 mg mL^−1^ isooctane solution (Sigma-Aldrich, Waltham, MA, USA). One mL of methanolic potassium hydroxide solution (0.4 mol L^−1^) was added to the lipid solution and maintained in a water bath at 100 °C for 10 min. A total of 3 mL of methanolic sulfuric acid solution (1 mol L^−1^) was added and the solution was maintained at 100 °C for 10 min in a water bath. The samples were immediately cooled, and 2 mL of isooctane was added to the samples. After phase separation, the upper layer containing fatty acid methyl esters (FAME) dissolved in isooctane was removed and subjected to chromatographic analysis.

The samples were analyzed on a GC-2010 Plus gas chromatograph coupled with a Flame Ionization Detector (FID) with an AOC-20is series autoinjector (Shimadzu, Kyoto, Japan). The column used was a Zebron ZB-Waxplus (60 m × 0.25 mm, film thickness 0.25 μm) with 100% polyethylene glycol from Phenomenex (Torrance, CA, USA). The carrier gas used was helium at a flow rate of 1.21 mL min^−1^. A volume of 1 μL of the sample was injected with a split ratio of 1:50. The injector temperature was maintained at 250 °C, the oven temperature was increased from 50 °C to 160 °C at a rate of 8 °C min^−1^, increasing the temperature at a rate of 5 °C min^−1^ to 240 °C (held for 25 min), and the detector temperature was maintained at 240 °C. The determination of fatty acids was performed using the method reported by Visentainer [10].

The FAME peaks were identified by comparing their retention times to those of the reference standards (Supelco 37 Component FAME Mix, Sigma-Aldrich, St. Louis, MO, USA) under the same conditions. Subsequently, the FAME peaks were quantified based on the FID correction factor between the analyte peak and methyl tricosanoate (Sigma-Aldrich, St. Louis, MO, USA), used as an internal standard, and the results were expressed in g of fatty acid per 100 g of lipid mass. Four replicates were performed for each sample.

#### 2.2.4. Acidity Index

Two grams of oil was added to a 125 mL Erlenmeyer flask with 25 mL of neutral ether/alcohol (2:1) solution. Afterward, two drops of 1% phenolphthalein alcoholic solution were added, and the sample was titrated with 0.1 M sodium hydroxide solution until it acquired a permanent pink color for 30 s. The index calculation was based on Equation (1).

Acidity index (%) = V × N × f × 28.2(1)

where

V—volume of sodium hydroxide (NaOH) used in the titration;
N—normality of the NaOH solution;
f—correction factor of the sodium hydroxide (NaOH) solution.

#### 2.2.5. Peroxide Index

Five grams of oil was added to a 500 mL Erlenmeyer flask with 30 mL of acetic acid/chloroform (3:2) solution. After stirring the sample, 0.5 mL saturated potassium iodide solution was added, and the sample was kept at rest for 1 min. After this time, 30 mL of water and 0.5 mL of 1% starch solution were added. The sample acquired a bluish color and was titrated with 0.01 M sodium thiosulfate solution until the blue color disappeared. The index calculation was based on Equation (2).

Peroxide index (meq O_2_ kg^−1^) = [(A − B) × N × f × 1000]/M (2)

where

A—volume of sodium thiosulfate used in the sample titration;
B—volume of sodium thiosulfate used in the blank titration;
N—normality of the sodium thiosulfate solution;
f—correction factor of the sodium thiosulfate solution;
M—mass of the sample.

#### 2.2.6. Iodine Index

A sample of 0.25 g of oil was added to a 500 mL Erlenmeyer flask with 20 mL of Wijs solution. The sample was shaken and left to stand for 30 min, protected from light. After this time, 10 mL of a 15% potassium iodide solution and 100 mL of freshly boiled and cooled water were added. Then, 2 mL of a 1% starch solution was added, and the sample was titrated with a 0.1 M sodium thiosulfate solution until the blue color disappeared. The index calculation was based on Equation (3).

Iodine index (g (100 g)^−1^) = [(VB − VA) × 0.1 × f × MMKI]/M (3)

where

VB—volume used in the blank titration;
VA—volume used in the sample titration;
f—sodium thiosulfate correction factor;
MMKI—molecular mass of potassium iodide;
M—mass of the sample.

#### 2.2.7. Saponification Index

Two grams of oil was added to a 500 mL Erlenmeyer flask with 20 mL of 4% potassium hydroxide alcoholic solution. The flask was heated for 30 min in a reflux refrigerator. After this time, the sample was removed and cooled. Two drops of 1% phenolphthalein solution were added and the sample was titrated with 0.5 M hydrochloric acid until the pink color disappeared. The index calculation was based on Equation (4).

Saponification index (mg KOH g^−1^) = 28.05 × f × (VB − VA)/M (4)

where

VA—volume used in sample titration;
VB—volume used in blank titration;
f—correction factor of the HCL solution;
M—mass of the sample.

#### 2.2.8. Protein

The nitrogen concentration in the samples was measured based on the methodology developed by Tedesco et al. [11]. It consisted of acid digestion (H_2_SO_4_ 0.025 mol L^−1^ + H_2_O_2_ 30% *v*/*v*) followed by distillation by the Kjeldahl method using a nitrogen distiller (SL-74, Solab, Piracicaba, Brazil). The following reagents were used in the acid digestion: potassium sulfate (Alphatec (Macaé, Brazil), 99.95%), copper II sulfate (ICO) (Dinâmica (Indaiatuba, Brazil), 98%), and sulfuric acid (Synth, 98%). The following reagents were used in the distillation: sodium hydroxide (Dinâmica, 98%), bromocresol green and methyl red indicators (Dinâmica), absolute ethyl alcohol (Dinâmica, 99.5%), hydrochloric acid (Alphatec, 37%), and boric acid (Alphatec, 99.5%). Four replicates were performed for each sample. The calculation was carried out based on the Kjeldahl method.

#### 2.2.9. Ash

Five grams of each sample was placed in crucibles and incinerated in a muffle furnace (2000-F, Zezimaq, Belo Horizonte, Brazil) at 550 °C for 2 h. The mass was measured on an analytical balance (M214Ai, Bel, Piracicaba, Brazil). Four replicates were performed for each sample.

#### 2.2.10. Carbohydrates

The determination of carbohydrates was determined by the difference through the “Nifext” fraction, calculated by the difference between 100% and the sum of the protein, lipid, moisture, and ash content.

#### 2.2.11. Nutrients

The quantification of the nutrients calcium (Ca), copper (Cu), iron (Fe), magnesium (Mg), manganese (Mn), potassium (K), phosphorus (P), sulfur (S) and zinc (Zn) was performed according to the methodology of Tedesco et al. [11] through acid digestion using a mixture of nitric acid and perchloric acid (HNO_3_ + HClO_4_, in the proportion of 3:1). The mixture was analyzed in an atomic absorption spectrometer (Perkin Elmer, Model AAnalyst 200, Waltham, MA, USA). For K, the readings were performed in a flame photometer (Digimed, Model DM62) and the elements P and S were determined by the colorimetric method in an atomic absorption spectrophotometer (Tecnal, Espec-UV-5100, Piracicaba, Brazil). For P and S, the readings were taken at wavelengths of 882 nm and 460 nm, respectively.

#### 2.2.12. Statistical Analysis

The quantitative responses were subjected to Analysis of Variance (ANOVA) using Minitab 18.0 software. For variables that presented significant effects at 95% confidence, the difference between means was analyzed using Tukey’s test, considering different mean values with *p*-value < 0.05.

## 3. Results

### 3.1. Moisture

The moisture ranged from 7.62 ± 0.26% for the R-S treatment to 8.92 ± 0.03% for the (C + R) − NS treatment in the crop year 21/22. In the crop year 22/23, the moisture varied from 11.02 ± 0.21% for the O − S treatment to 13.03 ± 0.83 for the R − S treatment (Table 2).

### 3.2. Grain Yield

**Figure 1:**
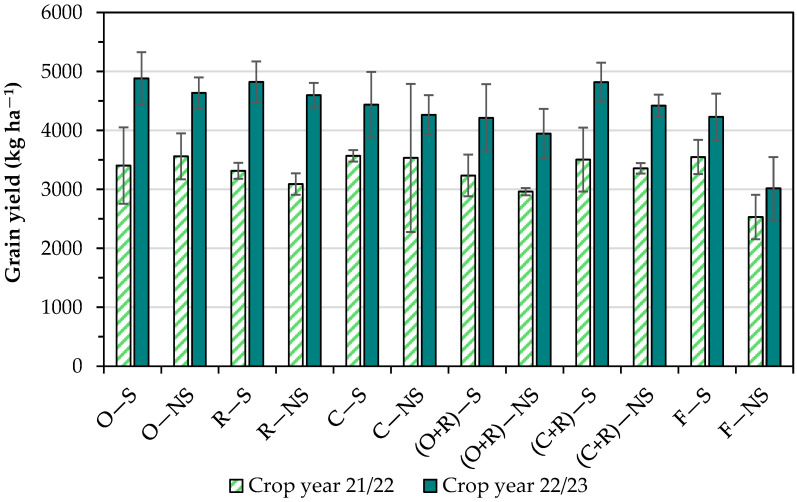
Yields of soybean grains grown in lowlands; error bars indicate the standard deviation.

The grain yield in soybean treatments grown in lowlands ranged from 2530.6 ± 378.8 kg ha^−1^ to 4881.8 ± 446.6 kg ha^−1^, with an average productivity of 3829.8 kg ha^−1^ (Figure 1). The following yields were obtained in three treatments: 2530.6 kg ha^−1^ in F − NS, 2962.6 kg ha^−1^ in (O + R) − NS, and 3089.8 kg ha^−1^ in R − NS. These grain yields occurred in the treatments with non-scarified soil. Conversely, the following yields were obtained in treatments in which the soil was scarified: 4881.8 kg ha^−1^ in O − S and 4823.2 kg ha^−1^ in R − S.

The results of the analysis of variance for grain yield showed that no factor was significant (*p* value > 0.05). In the crop year 21/22, the average grain yield was lower than crop year 22/23. This behavior can be justified by the water availability, which was lower in the crop year 21/22 because rainfall was lower, and no irrigation was used.

Even though no significant difference in the grain yield was achieved within each crop year, the use of cover crops seems to be favorable because the absolute values were larger than 3 and 4 tons ha^−1^ in 9/10 of treatments with cover crops in the crop years 21/22 and 22/23, respectively. Conversely, the absence of cover crops associated with no soil scarification (F − NS) presented grain yields near or smaller than 3 tons ha^−1^. For instance, according to the Brazilian National Supply Company (CONAB—https://www.conab.gov.br/info-agro/safras/graos/boletim-da-safra-de-graos, accessed on 27 October 2024), the average soybean yields in the crop years 21/22 and 22/23 in highland fields in Rio Grande do Sul State were 1.43 and 1.99 tons ha^−1^, respectively. For all states of Brazil, the average soybean yields in the crop years 21/22 and 22/23 in highland fields were 3.03 and 3.51 tons ha^−1^, respectively. Therefore, the results presented in this study regarding cultivation in lowlands indicate satisfactory results.

### 3.3. Quality Indicators

#### 3.3.1. Proteins

**Figure 2:**
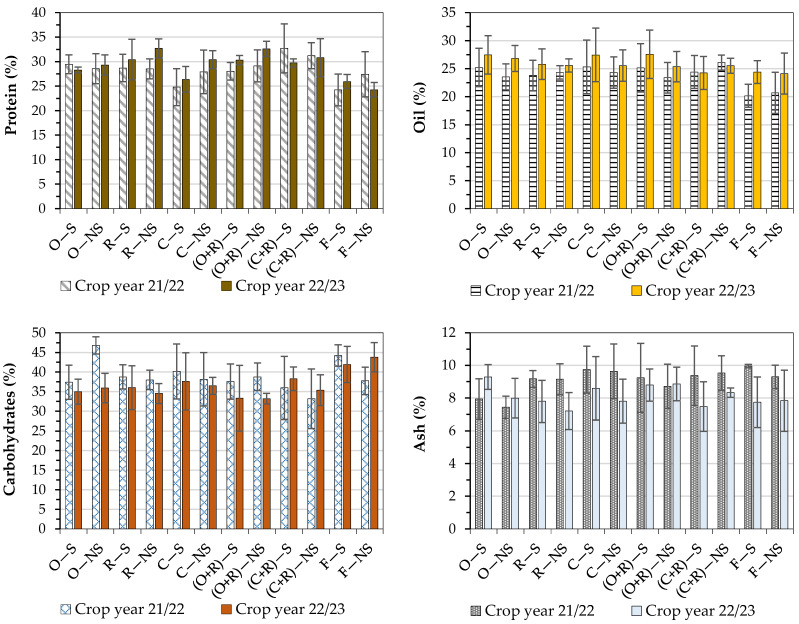
Total proteins, oil, carbohydrates, and ash in soybean grains obtained from different treatments in lowland fields; the values indicate the mean ± standard deviation (error bars).

The protein content ranged from 32.7% (mass basis) for the R − NS treatment to 24.2% for the F − S treatment (Figure 2). The ANOVA results for proteins in the crop year 21/22 showed that no significant difference (*p*-value > 0.05) was seen. For the crop year 22/23, the results were significant (*p*-value < 0.05). According to Tukey’s test at 95% confidence, higher significant values were obtained in treatments with the cover crop ryegrass, with some treatments differing from each other.

#### 3.3.2. Oil

The oil contents ranged from 27.6% (mass basis) for the (O + R) − S treatment to 20.2% in the F − S treatment (Figure 2). The ANOVA results for oil in both crop years showed that no significant difference (*p*-value > 0.05) was seen.

#### 3.3.3. Ash

The ash content ranged from 9.9% (mass basis) for the F − S treatment to 7.4% for the O − NS treatment (Figure 2). The ANOVA results in both crop years demonstrated that no significant difference (*p*-value > 0.05) was seen on ash.

#### 3.3.4. Carbohydrates

The carbohydrate content ranged from 46.8% (mass basis) for the O − NS treatment to 33.2% for the (C + R) − NS treatment (Figure 2). The ANOVA results in the crop year 21/22 showed that no factor was significant (*p*-value > 0.05). For the crop year 22/23, a significant difference (*p*-value < 0.05) was seen. Higher significant values were obtained in treatments with clover as a cover crop, with many treatments differing from each other. The variable “cover crop” yielded a significant difference (*p*-value < 0.05) in carbohydrate contents. Only the fallow treatment differed from oats, ryegrass, and ryegrass + oats.

#### 3.3.5. Fatty Acids in the Oil

The fatty acid composition of soybean oil in the treatments for the crop years 21/22 and 22/23 is presented in Table 3 and Table 4, respectively. The ANOVA results for fatty acids in the crop year 21/22 indicated no significant difference (*p*-value > 0.05). In the crop year 22/23, a statistical difference (Table 5) was seen for C15:0 (pentadecanoic acid), C18:3n3 (alpha-linolenic acid), and C20:3n6 (gamma-linolenic acid). Pentadecanoic acid (C15:0) presented the maximum value of 0.07% in the O—S treatment. Alpha-linolenic acid (C18:3n3) presented the maximum value of 5.86% in the treatment (O + R) − S (Table 5). As seen in Table 5, the variable “Cover crop” is significant (*p*-value < 0.05). Gamma linolenic acid (C20:3n6) presented the maximum value of 0.09% in the C − S treatment.

#### 3.3.6. Acidity Index of Oil

**Figure 3:**
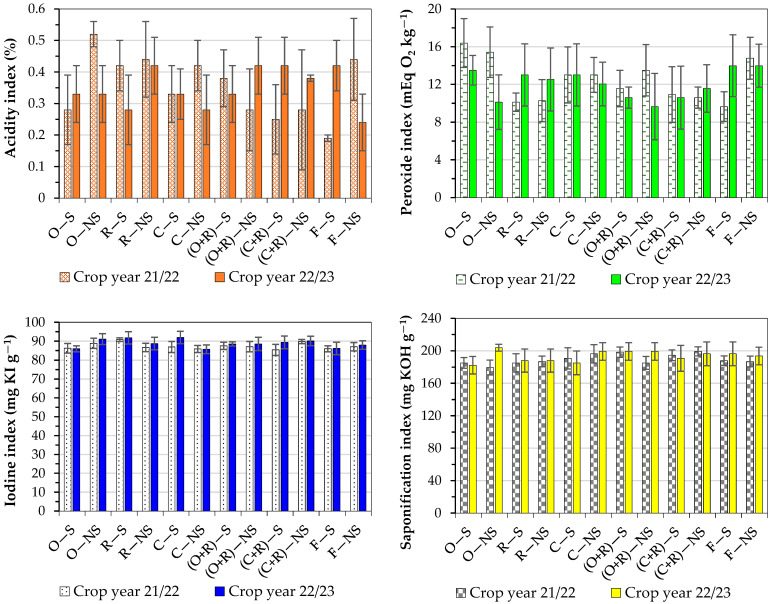
Acidity, peroxide, iodine, and saponification indices of soybean oil extracted from grains obtained from different treatments in lowland fields; the values indicate the mean ± standard deviation (error bars).

In the crop year 21/22, the acidity index ranged from 0.52% for the O − NS treatment to 0.19% for the F − S treatment. In the crop year 22/23, this index ranged from 0.42% for the R − NS treatment to 0.24% for the F − NS treatment (Figure 3). Typically, this value is found in the following range: 0.3 to 0.5% [12].

#### 3.3.7. Peroxide Index of Oil

In the crop year 21/22, the peroxide index ranged from 16.39 mEq O_2_ kg^−1^ for the O − S treatment to 10.12 mEq O_2_ kg^−1^ for the R − S treatment. In the crop year 22/23, this index ranged from 13.98 mEq O_2_ kg^−1^ for the F − S treatment to 9.64 mEq O_2_ kg^−1^ for the (O + R) − NS treatment (Figure 3). The Brazilian regulation establishes the following limiting value for refined vegetable oils: <15 mEq O_2_ kg^−1^ [13].

#### 3.3.8. Iodine Index of Oil

In the crop year 21/22, the iodine index ranged from 90.77 mg KI g^−1^ for the R − S treatment to 85.34 mg KI g^−1^ for the (C + R) − S treatment. In the crop year 22/23, this index ranged from 91.92 mg KI g^−1^ for the C − S treatment to 85.63 mg KI g^−1^ for the (C + R) − S treatment (Figure 3). The Brazilian regulation establishes the following limiting value for refined vegetable oils: >78 mg KI g^−1^ [13].

#### 3.3.9. Saponification Index of Oil

In the crop year 21/22, the saponification index ranged from 199.16 mg KOH g^−1^ for the (C + R) − NS treatment to 185.13 mg KOH g^−1^ for the O − S treatment. In the crop year 22/23, this index ranged from 203.74 mg KOH g^−1^ for the O − NS treatment to 182.33 mg KOH g^−1^ for the O − S treatment (Figure 3). The Brazilian regulation establishes the following limiting value for refined vegetable oils: >182 mg KOH g^−1^ [13].

#### 3.3.10. Nutrients

A significant difference between treatments was achieved for the exported quantities of N, K, Mg, and Zn in the soybean grains. The exported quantities of P, Ca, S, Cu, Fe, and Mn did not show any different behavior but presented quantities in the grains at satisfactory levels (Table 6).

The exported amounts of N in soybean grains in the treatments ranged from 37.87 to 46.27 kg ton^−1^. The treatments with ryegrass as a cover crop, alone or in consortia, yielded N of 46.27 kg ton^−1^ ((C + R) − NS) and 45.56 kg ton^−1^ ((O + R) − NS). Regarding the nutrient K, the double interaction “soil preparation” × “cover crop” was significant (*p*-value < 0.05). The highest K values were obtained in the treatments with ryegrass + oats in scarified soils. Regarding Mg, the variable “soil preparation” was significant (*p*-value < 0.05). The highest Mg values were obtained in scarified soils. For both K and Mg, the highest values were reached in treatments with scarified soil and ryegrass cover crops. The highest K value was 29.29 kg ton^−1^ in the (O + R) − S treatment and the highest Mg value was 2.57 kg ton^−1^ in the R − S treatment. The amounts of Zn ranged from 30.02 mg kg^−1^ to 38.0 mg kg^−1^, with the variable “soil preparation” being significant (*p*-value < 0.05), and the scarification provided samples with a higher amount of Zn.

## 4. Discussion

Satisfactory levels of grain yields were achieved, with an average yield of 3829.8 kg ha^−1^. In a previous study [7], the average yield of soybean grown in lowlands was 3697 kg ha^−1^. In that study, in a near field, the authors evaluated three factors, soil preparation, sowing season, and fertilization levels. According to the results presented in that paper, ranges in the grain yield in the lowlands were mainly affected by the sowing season and rainfall. According to Meert et al. [14], the Brazilian grain yield average for soybean cultivated in highlands is approximately 3300 hg ha^−1^. Therefore, our work demonstrated higher grain yields in approximately 70 and 90% of the treatments in crop years 21/22 and 22/23, respectively, which could be up to 46% higher than the aforementioned average.

The significant differences in soybean grain yield are linked to irregular rainfall patterns in several irrigated soybean growing areas. Lack of water stands out as one of the main reasons for this difference in grain yield in highland fields, especially in places where rainfall patterns are irregular. The lack of rainfall from December to March (which corresponds to the soybean growing season in Rio Grande do Sul), especially during years of La Niña conditions, is the main reason for the reduction in soybean grain yield in this Brazilian state [15].

Treatments with non-scarified soil tended to provide small grain yields. Otherwise, treatments in which the soil was scarified mostly provided high grain yields. In the crop year 21/22, the grain yields were lower compared to the crop year 22/23. This difference is explained by issues of water availability, which was lower in the crop year 21/22. The grain yield of soybean in highland fields is associated with soil moisture, especially at critical moments of their development throughout the growth phase [8]. Also, the potential productivity of soybean varies according to geographic location, which is influenced by climate change and environmental conditions. Climatic conditions are one of the main factors that impact the growth, development, and productive efficiency of soybean [16].

Both temperature and precipitation play key roles in the formation and maintenance of soybean reserves, and studies indicate that the oil level in soybean decreases when exposed to environmental stress conditions [7]. Another factor that affects grain yield is soil quality. In this context, cover crops play several essential roles in agricultural ecosystems and are valued for their contribution to soil improvement and their beneficial impact on its physical, chemical, and biological properties. They offer advantages such as weed control, nitrogen fixation, carbon capture, and increased productivity [17]. Knowledge about the physiology of the soybean grown in the lowlands is still limited. There is a need to evaluate the physical and chemical characteristics of soybean grown in these areas because indicators such as proteins and oil determine the quality of the grains. In this study, it was possible to observe that soybean grains generally have an oil percentage higher than 25 wt% in treatments that contained oat and ryegrass as a cover crop. Commonly, oat and ryegrass cover crops are considered the most widely used forage crops in crop rotation systems in rice cultivation areas. The practice of soil scarification helps to reduce flooding during rainy seasons and favors oxygen circulation, which can help in biological nitrogen fixation [7].

The considerable mass content of proteins and oil were obtained in soybean grains grown in lowland fields with different cover crops, in which fallow yielded lower mass values. In addition, high contents of oil were observed in samples from scarified soils. The highest protein values in soybean cultivated in 22/23 were associated with treatments with cover crops. When fallow was maintained in the autumn–winter season, a significant difference was seen, with lower protein values. Residues generated by cover crop production on the soil surface contribute to an increase in soil organic matter. Root exudates, located below the surface, diversify the energy sources for soil microorganisms, promoting the improvement of their characteristics. These characteristics benefit microbial communities, which increase the grain yield by increasing nutrient exportation [2]. Soybean contains approximately 35 wt% protein [18]. Although the exportation of nutrients provided higher grain yields in some treatments, the protein values in this work were slightly lower than the worldwide average, which can be attributed to the lower exportation of nitrogen.

Soybean is crucial in several industrial applications [19], especially in the production of biodiesel, because it is considered one of the main sources of oil for transesterification. With approximately 20 wt% oil and 35 wt% protein, soybean represents approximately 60% of the world’s supply of vegetable protein, making it one of the most important crops worldwide. In addition to field management practices, different processing conditions in the food industry, such as temperature and drying time, lead to different nutritional components and quality parameters. For example, a fluidized bed drying process was able to eliminate antinutritional factors, minimizing losses in soy flour quality. This drying process may be used before flour milling to avoid severe flour color changes [20].

Soybean belongs to the Fabaceae family and has a high nutrient content, including carbohydrates, lipids, proteins, minerals, fibers, and vitamins, in addition to having a low amount of saturated fats. They are especially rich in isoflavones (phytoestrogens) such as daidzein and genistein, which play an important role in biological activity, generating significant interest in the production of functional foods. For instance, soybean can be used to produce fermented beverages with 33% soybean milk [21].

Soybean oil is a mixture of the following five predominant fatty acids: palmitic, stearic, oleic, linoleic, and linolenic. The high presence of linoleic acid results in the low oxidative stability of the oil. The percentage of fatty acids in soybean oil varies, with 15% to 33% oleic acid, 43% to 56% linoleic acid, 5% to 11% linolenic acid, and 11% to 26% saturated acids. As a food product, the quality of the oil is largely determined by its fatty acid composition [18]. In this work, all the main fatty acids mentioned were in accordance with standard samples from the highlands.

Soybean contains all essential amino acids, such as isoleucine, histidine, leucine, lysine, methionine, phenylalanine, threonine, tryptophan, and valine, making it a highly nutritious crop. Therefore, various products are made from soybean, such as tofu, soy milk, soy sprouts, and soybean paste. Beyond the studies of management practices in fields, studies related to gene-based improvements to increase protein, oil, and amino acid content constitute a crucial goal in soybean breeding [22].

The amino acid and fatty acid profiles, together with the total amounts of oil and protein, are determinants of soybean quality. Soybean oil contains both unsaturated and saturated fatty acids. For human consumption, soybean oil with a higher content of monounsaturated fatty acids (24%) such as oleic acid and polyunsaturated fatty acids (60%) such as linolenic and linoleic are preferable to saturated fatty acids. A higher proportion of oleic acid and a lower proportion of linoleic and linolenic acid are preferred, as this increases the stability of the oil [23].

Regarding the composition of fatty acids in the crop year 21/22, the factors in the treatments were not significant in the values observed. In the crop year 22/23, a significant difference was seen for C15:0 (pentadecanoic acid), C18:3n3 (alpha-linolenic acid), and C20:3n6 (gamma-linolenic acid). For pentadecanoic acid and gamma-linolenic acid, the significant variable was soil preparation, with an increase in the presence of these acids in scarified soils. For alpha-linolenic acid, the significant variable was the cover crop. In the treatments in which ryegrass and oats were used as cover crops, an increase in this acid was noted. This outcome is important because polyunsaturated fatty acids are the most important nutritional components in edible oils or other functional foods [3].

Other soybean quality indicators were evaluated, including acidity, peroxides, iodine, and saponification indices. The acidity index indicates the state of conservation of the oil and typically varies between 0.3 and 0.5% for soybean [12]. Therefore, comparing the values of this work for crop year 21/22 with the values reported elsewhere in the literature, it can be observed that only the O − NS treatment was above the standard (>0.5%). In the crop year 22/23, all the treatments produced an acidity index lower than 0.5%. Three treatments were slightly below the minimum indicated, with the acidity in the range of 0.24–0.28%. Therefore, the results are in agreement with the need for commercialization and food production.

The peroxide index indicates the level of degradation of oils and fats. The oxidation rate depends on certain conditions, such as the presence of light, oxygen availability, and high temperatures. Auto-oxidation is based on a free radical mechanism, in which the absorption of oxygen causes the formation of hydroperoxides [24]. Values of this index lower than 15 mEq O_2_ kg^−1^ are within the range established by Brazilian regulations [13]. Therefore, most of the samples attained this maximum limit, with the treatments (O + R) − S, (C + R) − S, and (C + R) − NS presenting average values lower than 15 mEq O_2_ kg^−1^ for both crop years.

The iodine index measures the degree of unsaturation of fats extracted with ether. Consequently, the higher the unsaturation of the fatty acid, the higher its iodine absorption capacity and iodine index [25,26]. The National Health Surveillance Agency requires that vegetable oils have a maximum iodine index of 120 mg KI g^−1^ for commercialization, complying with the safety parameters for human consumption. According to Brazilian technical regulations, this index must have a minimum of 78 mg KI g^−1^ for oils with high oleic acid contents [13]. In this work, the values ranged from 85.3 to 91.9 mg KI g^−1^, which are in accordance with the aforementioned limits.

The saponification index indicates the mass of free fatty acids after saponification and the higher their average molecular weight, the lower the index value. This is more related to biodiesel production. The free fatty acids react and increase the solubility of the esters formed in the glycerol. This can interfere with the phase separation or cause the loss of esters in the separation of glycerol [27]. The Brazilian technical regulations establish this index in refined vegetable oils must have a minimum value of 182 mg KOH g^−1^. Therefore, the results found in this work meet this minimum limit.

The carbohydrate average content was approximately 37.8 and 36.7% in the crop years 21/22 and 22/23, respectively. These values are slightly higher than those reported for highland fields, which are approximately 30 wt% [28]. These higher values can be justified by the fact that the sum of the other components, especially total proteins, was lower in the lowlands. Even though the results presented in this work are lower than those presented by a study performed with soybean grown in the lowlands with different fertilization levels, which were approximately 38% on average [7], this indicates more studies are necessary on this subject to optimize soybean cultivation.

A large amount of ash is undesirable in the food industry because it can affect the quality of soy products or reduce the yield of target compounds. Ash contains inorganic components consisting of nutritional elements provided in the fertilization of the crop, such as Mn [7]. Some nutrients in the soils of lowland fields were exported to the grains at high levels, which may have contributed to the increase in ash levels in the crop years studied.

Otherwise, the exported amounts of N in the soybean grains in the lowland treatments were low, ranging from 37.87 to 46.27 kg ton^−1^. It is estimated that 80 kg of nitrogen is needed to produce one ton of grains. Given this, the inclusion of cover crops improves the soil’s physical properties and microbial enzyme activities. They maintain the soil moisture state, influencing evaporation, drainage, and runoff after rain events [8]. N is one of the nutrients most required by the soybean crop, with ryegrass as a cover crop providing higher levels of N. This is an important result because the use of cover crops as a management practice has been adopted in many no-tillage systems, which can be improved by future studies with the publication of more findings to the scientific community.

For both K and Mg, the treatments with scarified soil and ryegrass as cover crops indicated high levels in soybean grains. This can be explained because soil scarification allows a reduction in waterlogging during periods with high precipitation and an increase in oxygen diffusion, which can benefit biological nitrogen fixation [7]. Another factor that influences the amount of nutrients available to grains is cover crops, as they increase the availability of phosphorus, potassium, nitrogen, and other macro- and micronutrients for subsequent crops. As observed in the study, treatments with cover crops, mainly ryegrass and oats, resulted in better grain qualities, with a higher amount of nutrients.

## 5. Conclusions

The quality indicators of soybean cultivated in lowland fields indicated that it has physical-chemical characteristics to be applied in the food industry and other chemical-related industries. The grain yields were mostly higher than the averages in traditional highland fields, which is an important economic outcome for commercialization. Soil scarification positively influences grain yield and contributes to high protein and oil content. Regarding the oil content, it was on average 23.9 and 25.8% higher for crop years 21/22 and 22/23, which is approximately 1.2 times higher than the content found in highland fields. The oil presented fatty acids and quality indices (acidity, peroxide, iodine, and saponification) within the ranges established by Brazilian regulations for refined vegetable oils. The only parameter that could be higher was protein, which remained approximately 0.8–0.9-fold the average found in highlands. Thus, the conditions tested in terms of fertilization should be revised and improved, especially to increase the exportation of nitrogen. For nutrients, soil scarification allows for higher levels of absorption by the soybean. Regarding nitrogen, the use of ryegrass as a cover crop allows for higher levels of absorption. Therefore, as a guide for novel investigations into large-scale applications in the future, the use of ryegrass as a cover crop and soil scarification are recommended.

## Figures and Tables

**Table 1:** Codification of treatments.

Code	Meaning
O − S	Oat (cover crop)—Scarified (soil preparation)
O − NS	Oat (cover crop)—Non-scarified (soil preparation)
R − S	Ryegrass (cover crop)—Scarified (soil preparation)
R − NS	Ryegrass (cover crop)—Non-scarified (soil preparation)
C − S	Clover (cover crop)—Scarified (soil preparation)
C − NS	Clover (cover crop)—Non-scarified (soil preparation)
(O + R) − S	Oat + Ryegrass (cover crop)—Scarified (soil preparation)
(O + R) − NS	Oat + Ryegrass (cover crop)—Non-scarified (soil preparation)
(C + R) − S	Clover + Ryegrass (cover crop)—Scarified (soil preparation)
(C + R) − NS	Clover + Ryegrass (cover crop)—Non-scarified (soil preparation)
F − S	Fallow (control)—Scarified (soil preparation)
F − NS	Fallow (control)—Non-scarified (soil preparation)

**Table 2:** Moisture of soybean grown in lowlands in the crop years 21/22 and 22/23.

Treatment	Crop Year 21/22	Crop Year 22/23
	Moisture (%)	
O − S	8.14 ± 0.79	11.02 ± 0.21
O − NS	8.04 ± 0.55	11.75 ± 1.44
R − S	7.62 ± 0.26	13.03 ± 0.83
R − NS	8.64 ± 0.85	11.59 ± 1.65
C − S	7.92 ± 0.54	11.55 ± 1.27
C − NS	8.71 ± 0.65	11.57 ± 1.10
(O + R) − S	8.26 ± 0.92	12.05 ± 1.10
(O + R) − NS	8.58 ± 0.64	10.70 ± 0.53
(C + R) − S	8.72 ± 0.33	12.26 ± 0.59
(C + R) − NS	8.92 ± 0.03	11.41 ± 1.01
F − S	7.67 ± 0.40	11.02 ± 0.30
F − NS	8.03 ± 0.51	11.60 ± 1.06

**Table 3:** Fatty acids in soybean oil extracted from soybean subjected to different treatments in lowland fields in the crop year 21/22.

Treatment	Fatty Acids (%) *				
	C14:0	C15:0	C16:0	C16:1	C17:0
O − S	0.09 ± 0.01	0.01 ± 0.01	11.70 ± 0.34	0.09 ± 0.01	0.06 ± 0.01
O − NS	0.10 ± 0.05	0.01 ± 0.01	13.07 ± 0.17	0.08 ± 0.01	0.08 ± 0.01
R − S	0.14 ± 0.02	0.02 ± 0.01	11.55 ± 0.39	0.09 ± 0.02	0.08 ± 0.01
R − NS	0.09 ± 0.01	0.01 ± 0.01	14.85 ± 0.82	0.10 ± 0.05	0.10 ± 0.02
C − S	0.09 ± 0.03	0.02 ± 0.01	14.98 ± 0.32	0.08 ± 0.02	0.10 ± 0.02
C − NS	0.08 ± 0.01	0.03 ± 0.01	12.58 ± 1.10	0.07 ± 0.01	0.09 ± 0.01
(O + R) − S	0.08 ± 0.01	0.02 ± 0.01	14.74 ± 1.07	0.06 ± 0.02	0.06 ± 0.02
(O + R) − NS	0.08 ± 0.01	0.01 ± 0.01	17.10 ± 1.23	0.10 ± 0.03	0.09 ± 0.01
(C + R) − S	0.08 ± 0.01	0.06 ± 0.02	12.36 ± 0.54	0.07 ± 0.01	0.08 ± 0.01
(C + R) − NS	0.14 ± 0.06	0.01 ± 0.01	19.53 ± 1.94	0.11 ± 0.06	0.12 ± 0.03
F − S	0.24 ± 0.04	0.05 ± 0.02	17.72 ± 1.22	0.08 ± 0.02	0.09 ± 0.02
F − NS	0.07 ± 0.01	0.03 ± 0.01	12.51 ± 1.28	0.08 ± 0.01	0.08 ± 0.01
**Treatment**	**C18:0**	**C18:1n9 cis/trans**	**C18:2n6 cis/trans**	**C18:3n3**	**C20:0**
O − S	3.98 ± 0.60	23.79 ± 1.80	52.18 ± 1.58	6.23 ± 0.77	0.38 ± 0.03
O − NS	4.85 ± 1.37	24.74 ± 2.56	49.44 ± 1.53	5.59 ± 1.47	0.64 ± 0.07
R − S	4.11 ± 0.54	24.75 ± 0.62	51.82 ± 1.22	5.99 ± 0.44	0.37 ± 0.11
R − NS	5.34 ± 1.25	25.47 ± 1.64	46.28 ± 1.93	5.05 ± 0.91	0.81 ± 0.05
C − S	5.41 ± 1.01	23.46 ± 2.74	45.62 ± 2.07	4.74 ± 0.42	0.83 ± 0.06
C − NS	4.82 ± 0.23	25.67 ± 1.23	49.52 ± 1.76	5.21 ± 0.56	0.53 ± 0.13
(O + R) − S	5.42 ± 1.00	24.76 ± 2.10	47.16 ± 1.83	4.89 ± 0.68	0.79 ± 0.14
(O + R) − NS	6.00 ± 1.31	23.62 ± 2.48	42.70 ± 2.03	4.63 ± 0.94	1.27 ± 0.12
(C + R) − S	4.47 ± 0.38	24.95 ± 2.32	48.96 ± 1.98	4.46 ± 1.87	0.38 ± 0.04
(C + R) − NS	6.29 ± 1.88	23.89 ± 1.84	40.68 ± 2.36	4.95 ± 1.10	1.73 ± 0.13
F − S	5.62 ± 1.87	23.23 ± 2.52	45.34 ± 2.23	5.03 ± 1.54	1.10 ± 0.18
F − NS	4.42 ± 0.36	24.94 ± 1.45	49.32 ± 2.21	5.42 ± 0.32	0.42 ± 0.07
**Treatment**	**C20:1n9**	**C20:3n6**	**C20:5n3**	**C22:0**	**C24:0**
O − S	0.16 ± 0.03	0.05 ± 0.01	0.35 ± 0.05	0.57 ± 0.10	0.15 ± 0.01
O − NS	0.19 ± 0.05	0.03 ± 0.01	0.40 ± 0.15	0.52 ± 0.12	0.21 ± 0.07
R − S	0.15 ± 0.05	0.02 ± 0.01	0.30 ± 0.10	0.40 ± 0.08	0.17 ± 0.02
R − NS	0.21 ± 0.04	0.04 ± 0.01	0.80 ± 0.10	0.53 ± 0.05	0.25 ± 0.01
C − S	0.20 ± 0.04	0.03 ± 0.01	3.53 ± 0.13	0.50 ± 0.04	0.27 ± 0.09
C − NS	0.19 ± 0.01	0.03 ± 0.01	0.17 ± 0.10	0.60 ± 0.11	0.30 ± 0.07
(O + R) − S	0.20 ± 0.02	0.02 ± 0.02	0.31 ± 0.01	1.03 ± 0.03	0.23 ± 0.01
(O + R) − NS	0.21 ± 0.03	0.06 ± 0.03	0.57 ± 0.03	2.56 ± 0.16	0.31 ± 0.08
(C + R) − S	0.17 ± 0.03	0.02 ± 0.01	0.36 ± 0.08	0.42 ± 0.05	0.22 ± 0.07
(C + R) − NS	0.25 ± 0.08	0.11 ± 0.01	0.95 ± 0.11	0.74 ± 0.02	0.34 ± 0.09
F − S	0.23 ± 0.10	0.05 ± 0.01	0.28 ± 0.06	0.65 ± 0.05	0.28 ± 0.06
F − NS	0.17 ± 0.03	0.02 ± 0.01	0.66 ± 0.06	0.40 ± 0.15	0.19 ± 0.05

* Mean ± standard deviation (n = 4). C14:0—Myristic acid; C15:0—Pentadecanoic acid; C16:0—Palmitic acid; C16:1—Palmitoleic acid; C17:0—Heptadecanoic acid; C18:0—Stearic acid; C18:1n9—Oleic acid; C18:2n6—Linoleic acid; C18:3n3—Alpha-Linolenic acid; C20:0—Alpha-Linolenic acid; C20:1n9—cis-Eicosenoic acid; C20:3n6—Gamma-Linolenic acid; C20:5n3—Eicosapentaenoic acid; C22:0—Behenic acid; C24:0—Lignoceric acid.

**Table 4:** Fatty acids in soybean oil extracted from soybean subjected to different treatments in lowland fields in the crop year 22/23.

Treatment	Fatty Acids (%) *				
	C14:0	C15:0	C16:0	C16:1	C17:0
O − S	0.12 ± 0.04	0.07 ± 0.01	16.18 ± 1.04	0.10 ± 0.03	0.10 ± 0.02
O − NS	0.14 ± 0.02	0.00 ± 0.00	12.02 ± 1.25	0.10 ± 0.06	0.09 ± 0.02
R − S	0.08 ± 0.01	0.02 ± 0.01	11.62 ± 0.37	0.08 ± 0.01	0.06 ± 0.01
R − NS	0.09 ± 0.01	0.02 ± 0.01	11.61 ± 0.36	0.08 ± 0.01	0.08 ± 0.01
C − S	0.11 ± 0.02	0.04 ± 0.01	21.86 ± 1.46	0.10 ± 0.06	0.12 ± 0.02
C − NS	0.08 ± 0.01	0.02 ± 0.01	17.57 ± 1.19	0.06 ± 0.01	0.06 ± 0.01
(O + R) − S	0.11 ± 0.05	0.02 ± 0.01	15.53 ± 1.06	0.10 ± 0.03	0.09 ± 0.02
(O + R) − NS	0.23 ± 0.03	0.02 ± 0.01	11.83 ± 0.58	0.08 ± 0.01	0.08 ± 0.01
(C + R) − S	0.08 ± 0.01	0.02 ± 0.01	13.32 ± 1.98	0.07 ± 0.01	0.09 ± 0.01
(C + R) − NS	0.08 ± 0.01	0.02 ± 0.01	12.72 ± 1.49	0.07 ± 0.01	0.08 ± 0.01
F − S	0.08 ± 0.01	0.02 ± 0.01	15.61 ± 1.99	0.08 ± 0.01	0.09 ± 0.02
F − NS	0.08 ± 0.01	0.02 ± 0.01	12.82 ± 1.44	0.08 ± 0.01	0.08 ± 0.01
**Treatment**	**C18:0**	**C18:1n9 cis/trans**	**C18:2n6 cis/trans**	**C18:3n3**	**C20:0**
O − S	5.36 ± 1.07	22.86 ± 1.87	44.38 ± 2.96	5.20 ± 0.60	1.08 ± 0.18
O − NS	4.44 ± 0.75	24.92 ± 2.20	48.28 ± 3.95	5.36 ± 0.90	0.45 ± 0.18
R − S	4.45 ± 0.97	26.00 ± 1.59	50.08 ± 3.20	5.64 ± 1.25	0.44 ± 0.07
R − NS	4.39 ± 0.19	24.76 ± 2.52	51.00 ± 1.71	5.67 ± 0.38	0.45 ± 0.05
C − S	7.12 ± 1.42	24.79 ± 1.02	36.32 ± 1.57	3.84 ± 1.34	1.95 ± 0.15
C − NS	5.78 ± 1.17	23.07 ± 0.85	43.52 ± 2.92	3.68 ± 1.39	1.04 ± 0.28
(O + R) − S	4.86 ± 1.50	22.13 ± 1.48	49.18 ± 2.32	5.86 ± 1.29	0.77 ± 0.16
(O + R) − NS	4.36 ± 0.87	24.43 ± 2.08	51.49 ± 2.71	5.78 ± 1.09	0.39 ± 0.11
(C + R) − S	5.16 ± 1.28	25.75 ± 1.32	48.25 ± 2.72	5.04 ± 1.00	0.70 ± 0.16
(C + R) − NS	5.07 ± 0.58	26.32 ± 0.98	48.56 ± 1.94	4.92 ± 0.60	0.60 ± 0.11
F − S	5.23 ± 1.56	24.31 ± 1.82	47.15 ± 2.19	5.35 ± 1.20	0.86 ± 0.10
F − NS	4.51 ± 0.65	23.93 ± 1.47	50.83 ± 2.87	5.86 ± 0.57	0.52 ± 0.17
**Treatment**	**C20:1n9**	**C20:3n6**	**C20:5n3**	**C22:0**	**C24:0**
O − S	0.17 ± 0.07	0.07 ± 0.01	1.18 ± 0.08	2.24 ± 0.14	0.16 ± 0.02
O − NS	0.18 ± 0.07	0.00 ± 0.00	3.33 ± 0.23	0.43 ± 0.11	0.24 ± 0.06
R − S	0.18 ± 0.04	0.05 ± 0.01	0.29 ± 0.07	0.63 ± 0.16	0.18 ± 0.02
R − NS	0.17 ± 0.03	0.02 ± 0.01	0.80 ± 0.09	0.54 ± 0.03	0.28 ± 0.01
C − S	0.27 ± 0.07	0.09 ± 0.02	0.86 ± 0.06	0.87 ± 0.16	0.36 ± 0.06
C − NS	0.21 ± 0.04	0.03 ± 0.01	0.32 ± 0.07	0.63 ± 0.05	0.29 ± 0.03
(O + R) − S	0.18 ± 0.03	0.04 ± 0.01	0.23 ± 0.07	0.53 ± 0.07	0.25 ± 0.09
(O + R) − NS	0.17 ± 0.05	0.02 ± 0.01	0.45 ± 0.13	0.43 ± 0.07	0.20 ± 0.01
(C + R) − S	0.21 ± 0.04	0.04 ± 0.01	0.35 ± 0.02	0.60 ± 0.03	0.28 ± 0.02
(C + R) − NS	0.20 ± 0.01	0.03 ± 0.01	0.37 ± 0.08	0.63 ± 0.07	0.30 ± 0.02
F − S	0.22 ± 0.08	0.04 ± 0.01	0.17 ± 0.09	0.54 ± 0.07	0.22 ± 0.01
F − NS	0.17 ± 0.01	0.03 ± 0.01	0.34 ± 0.04	0.47 ± 0.10	0.22 ± 0.05

* Mean ± standard deviation (n = 4). C14:0—Myristic acid; C15:0—Pentadecanoic acid; C16:0—Palmitic acid; C16:1—Palmitoleic acid; C17:0—Heptadecanoic acid; C18:0—Stearic acid; C18:1n9—Oleic acid; C18:2n6—Linoleic acid; C18:3n3—Alpha-Linolenic acid; C20:0—Alpha-Linolenic acid; C20:1n9—cis-Eicosenoic acid; C20:3n6—Gamma-Linolenic acid; C20:5n3—Eicosapentaenoic acid; C22:0—Behenic acid; C24:0—Lignoceric acid.

**Table 5:** Comparisons through Tukey’s test of significant variables on total proteins, carbohydrates, and fatty acids for the samples from the crop year 22/23.

**Total Protein**				
**Cover Crop**	**N**	**Average**	Grouping *	
R	8	31.6	A	
O + R	8	31.5	A	
C + O	8	30.3	A	
O	8	28.8	A	
C	8	28.4	A	B
F	8	25.1		B
**Carbohydrates**				
**Cover Crop**	**N**	**Average**	Grouping *	
F	8	42.9	A	
C	8	37.0	A	B
C + R	8	36.8	A	B
O	8	35.4		B
R	8	35.3		B
O + R	8	33.3		B
**C15:0 Fatty Acid**				
**Soil Preparation**	**N**	**Average**	Grouping *	
S	24	0.03	A	
NS	24	0.02		B
**C18:3n3 Fatty Acid**				
**Cover Crop**	**N**	**Average**	Grouping *	
O + R	8	5.82	A	
R	8	5.65	A	B
F	8	5.60	A	B
O	8	5.28	A	B
C + R	8	4.98	A	B
C	8	3.76		B
**C20:3n6 Fatty Acid**				
**Soil Preparation**	**N**	**Average**	Grouping *	
S	24	0.06	A	
NS	24	0.02		B

* Means followed by the same letter for each response do not statistically differ from each other using the Tukey test at a 95% confidence level; N: number of samples.

**Table 6:** Nutrients in soybean grains obtained in different treatments in lowland fields.

Treatment	N	P	K	Ca	Mg	S	Zn	Cu	Fe	Mn
	kg ton^−1^						g ton^−1^			
O − S	42.51 ± 2.60	5.70 ± 0.36	27.13 ± 1.72	2.96 ± 0.32	2.37 ± 0.14	2.93 ± 0.24	38.00 ± 1.03	10.76 ± 1.33	118.53 ± 6.95	44.23 ± 2.96
O − NS	37.90 ± 11.27	5.58 ± 0.56	19.41 ± 0.62	3.03 ± 0.21	2.41 ± 0.18	3.11 ± 0.29	37.05 ± 1.36	11.68 ± 1.07	122.02 ± 5.00	45.20 ± 2.87
R − S	43.32 ± 4.80	5.22 ± 0.75	19.73 ± 0.98	3.01 ± 0.27	2.57 ± 0.13	2.93 ± 0.22	36.46 ± 1.57	10.0 ± 1.14	120.30 ± 5.65	43.92 ± 1.13
R − NS	44.93 ± 2.74	5.60 ± 0.53	18.87 ± 0.38	2.85 ± 0.22	2.42 ± 0.23	3.18 ± 0.29	35.54 ± 2.63	10.55 ± 1.10	116.95 ± 8.67	45.99 ± 3.34
C − S	37.87 ± 4.46	5.12 ± 0.39	19.42 ± 0.78	2.98 ± 0.19	2.47 ± 0.21	2.85 ± 0.18	37.21 ± 1.43	10.94 ± 1.55	111.42 ± 6.86	43.83 ± 2.35
C − NS	42.87 ± 4.63	5.47 ± 0.50	19.45 ± 0.97	3.02 ± 0.28	2.41 ± 0.17	2.99 ± 0.17	35.18 ± 2.48	11.18 ± 0.90	119.02 ± 6.01	43.58 ± 3.34
(O + R) − S	42.88 ± 2.17	5.40 ± 0.66	29.29 ± 1.44	2.90 ± 0.27	2.51 ± 0.13	3.12 ± 0.26	36.75 ± 3.07	10.97 ± 1.60	116.39 ± 9.46	44.45 ± 2.09
(O + R) − NS	45.56 ± 3.32	5.47 ± 0.67	19.16 ± 0.68	2.79 ± 0.19	2.45 ± 022	3.05 ± 0.32	35.52 ± 2.59	9.73 ± 1.42	118.15 ± 6.65	47.08 ± 2.74
(C + R) − S	43.95 ± 5.15	5.54 ± 0.43	19.36 ± 0.79	2.88 ± 0.18	2.44 ± 0.14	3.19 ± 0.20	34.95 ± 1.82	10.31 ± 1.66	116.57 ± 5.24	45.85 ± 2.43
(C + R) − NS	46.27 ± 3.40	5.48 ± 0.53	19.60 ± 0.69	3.05 ± 0.18	2.41 ± 0.20	3.12 ± 0.31	36.15 ± 1.37	11.26 ± 1.43	117.97 ± 7.87	44.58 ± 3.02
F − S	38.40 ± 3.97	5.56 ± 0.40	19.70 ± 0.70	3.00 ± 0.27	2.48 ± 013	3.11 ± 0.20	36.36 ± 2.57	10.94 ± 1.05	116.94 ± 9.12	43.98 ± 3.41
F − NS	42.96 ± 8.46	5.79 ± 0.37	19.68 ± 0.76	2.89 ± 0.28	2.43 ± 0.17	3.05 ± 0.36	30.02 ± 2.45	11.29 ± 1.43	117.74 ± 10.22	44.25 ± 2.49

## Data Availability

The original contributions presented in the study are included in the article, further inquiries can be directed to the corresponding author.

## References

[1] Jung J.W., Park S.Y., Oh S.D., Jang Y., Suh S.J., Park S.K., Ha S.H., Kim J.K. (2022). Metabolomic Variability of Different Soybean Genotypes: β-Carotene-Enhanced (*Glycine max*), Wild (*Glycine soja*), and Hybrid (*Glycine max* × *Glycine soja*) Soybeans. Foods.

[2] Oilseeds: World Markets and Trade—United States Department of Agriculture. https://apps.fas.usda.gov/psdonline/circulars/oilseeds.pdf.

[3] Quédraogo E.R., Konaté K., Sanou A., Sama H., Compaoré E.W.R., Sytar O., Hilou A., Brestic M., Dicko M.H. (2022). Assessing the Quality of Burkina Faso Soybeans Based on Fatty Acid Composition and Pesticide Residue Contamination. Molecules.

[4] Szpunar-Krok E., Wondołowska-Grabowska A. (2022). Quality Evaluation Indices for Soybean Oil in Relation to Cultivar, Application of N Fertiliser and Seed Inoculation with *Bradyrhizobium japonicum*. Foods.

[5] Deng L. (2021). Current Progress in the Utilization of Soy-Based Emulsifiers in Food Applications—A Review. Foods.

[6] Jung K.C., Kim B.Y., Kim M.J., Kim N.K., Kim Y.H., Park H.M., Jang H.S., Shin H.C., Kim T.J. (2023). Development of a Gene-Based Soybean-Origin Discrimination Method Using Allele-Specific Polymerase Chain Reaction. Foods.

[7] Ody L.P., Baisch J.S., Ugalde G., Grohs M., Dorneles A.B., Neu G.R.F., Santos M.S.N., Ferreira P.A.A., Tres M.V., Zabot G.L. (2024). Early Sowing and Soil Scarification Improve Protein and Oil Contents in Soybean Grains Cultivated in Lowlands. J. Soil Sci. Plant Nutr..

[8] Pokhrel S., Kingery W.L., Cox M.S., Shankle M.W., Shanmugam S.G. (2021). Impact of Cover Crops and Poultry Litter on Selected Soil Properties and Yield in Dryland Soybean Production. Agronomy.

[9] Confortin T.C., Todero I., Luft L., Ugalde G.A., Mazutti M.A., Oliveira Z.B., Bottega E.L., Knies A.E., Zabot G.L., Tres M.V. (2019). Oil yields, protein contents, and cost of manufacturing of oil obtained from different hybrids and sowing dates of canola. J. Environ. Chem. Eng..

[10] Visentainer J.V. (2012). Analytical aspects of the fame ionization detector response of fatty acid esters in biodiesels and foods. Quim. Nova.

[11] Tedesco M., Gianello C., Bissani C.A., Bohnen H., VolkWeiss S.J. (1995). Análises de solo, plantas e outros materiais. Bol. Tec. Solos.

[12] Oliveira M.A., Lorini I., Mandarino J.M.G., Benassi V.T., França-Neto J.B., Henning A.A., Krzyzanowski F.C., Henning F.A., Hirakuri M.H., Leite R.S. Índice de acidez titulável dos grãos de soja colhidos nas safras 2015/2016 e 2016/17 no Brasil. Proceedings of the VIII Congresso Brasileiro de Soja.

[13] Instrução Normativa 49/2006—Ministério da Agricultura, Pecuária e Abastecimento. https://sistemasweb.agricultura.gov.br/sislegis/action/detalhaAto.do?method=visualizarAtoPortalMapa&chave=643062246.

[14] Meert L., Fernandes F.B., Müller M.M., Rizzardi D.A., Espindola J.S. (2020). Coinoculation with *Bradyrhizobium japonicum* and *Azospirillum brasilense* on soybean crop. Agron. Crop J..

[15] Mulazzani R.P., Boeno D., Ribeiro B.S., Alves A.F., Zanon A.J., Gubiani P.I. (2024). Chemical constraints are the major limiting factor of root deepening in southern Brazil soils. Geoderma Reg..

[16] Toleikiene M., Slepetys J., Sarunaite L., Lazauskas S., Deveikyte I., Kadziuliene Z. (2021). Soybean Development and Productivity in Response to Organic Management above the Northern Boundary of Soybean Distribution in Europe. Agronomy.

[17] Grün E., Alves A.F., Silva A.L., Zanon A.J., Corrêa A.R., Leichtweis E.M., Neto R.C., Ulguim A.R. (2024). How Do Off-Season Cover Crops Affect Soybean Weed Communities?. Agriculture.

[18] Wang J., Hong H., Yan X., Nan J., Lu Q., Gu Y., Qiu L. (2024). Stability Evaluation for Main Quality Traits of Soybean in the Northeast and Huang-Huai-Hai Regions. Agronomy.

[19] Ramos P.R., Rodrigues L.C., Zabot G.L., Oliveira A.L. (2024). Extraction of soybean oil with pressurized ethanol: Prospects for a new processing approach with analysis of the physical properties of crude oil and implementation costs through scale-up in an intermittent process. Processes.

[20] Martínez M.L., Marín M.A., Ribotta P.D. (2013). Optimization of soybean heat-treating using a fluidized bed dryer. J. Food Sci. Technol..

[21] Domínguez-Murillo A.C., Urías-Silvas J.E. (2024). Plant-based milk substitutes as probiotic vehicles: Health effect and survival, a review. Food Chem. Adv..

[22] Di Q., Piersanti A., Zhang Q., Miceli C., Li H., Liu X. (2022). Genome-Wide Association Study Identifies Candidate Genes Related to the Linoleic Acid Content in Soybean Seeds. Int. J. Mol. Sci..

[23] Kim W.G., Kang B.H., Kang S., Shin S., Chowdhury S., Jeong S.C., Choi M.S., Park S.K., Moon J.K., Ryu J. (2023). A Genome-Wide Association Study of Protein, Oil, and Amino Acid Content in Wild Soybean (*Glycine soja*). Plants.

[24] Oliveira M.B., Pogorzelski E.S., Pfeifemberg R., Knies A.E., Oliveira Z.B., Santos M.S.N., Zabot G.L., Tres M.V. (2024). Analysis of the cultivation of canola hybrids at different sowing dates. Acta Sci..

[25] Cubukcu P. (2023). Determination oil and fatty acid profiles of selected soybean (*Glycine max* L.) cultivars under second crop condition in eats Mediterranean agroecology. Turkish J. Field Crops.

[26] Arab R., Casal S., Pinho T., Cruz R., Freidja M.L., Lorenzo J.M., Hano C., Madani K., Boulekbache-Makhlouf L. (2022). Effects of Seed Roasting Temperature on Sesame Oil Fatty Acid Composition, Lignan, Sterol and Tocopherol Contents, Oxidative Stability and Antioxidant Potential for Food Applications. Molecules.

[27] Pasha M.K., Dai L., Liu D., Guo M., Du W. (2021). An overview to process design, simulation and sustainability evaluation of biodiesel production. Biotechnol. Biofuels Bioprod..

[28] Fonseca A.F., Neves E., Dipple F.L., Lima T.C. (2020). Correlation between productivity and centesimal composition of soybean cultivars. Sci. Electron. Arch..

